# Differences in the Cortical Structure of the Whole Fibula and Tibia Between Long-Distance Runners and Untrained Controls. Toward a Wider Conception of the Biomechanical Regulation of Cortical Bone Structure

**DOI:** 10.3389/fendo.2019.00833

**Published:** 2019-11-27

**Authors:** Sergio H. Lüscher, Laura M. Nocciolino, Nicolás Pilot, Leonardo Pisani, Alex Ireland, Jörn Rittweger, José L. Ferretti, Gustavo R. Cointry, Ricardo F. Capozza

**Affiliations:** ^1^Center for P-Ca Metabolism Studies (CEMFoC), National University of Rosario, Rosario, Argentina; ^2^Unity of Musculoskeletal Biomechanical Studies (UDEBOM), Universidad del Gran Rosario, Rosario, Argentina; ^3^School of Healthcare Science, Manchester Metropolitan University, Manchester, United Kingdom; ^4^Institute of Aerospace Medicine, German Aerospace Center (DLR), Cologne, Germany; ^5^Department of Pediatrics and Adolescent Medicine, University of Cologne, Cologne, Germany

**Keywords:** fibula, bone biomechanics, pQCT, running, bone mechanostat, exercise and bone

## Abstract

The cortical structure of human fibula varies widely throughout the bone suggesting a more selective adaptation to different mechanical environments with respect to the adjacent tibia. To test this hypothesis, serial-pQCT scans of the dominant fibulae and tibiae of 15/15 men/women chronically trained in long-distance running were compared with those of 15/15 untrained controls. When compared to controls, the fibulae of trained individuals had similar (distally) or lower (proximally) cortical area, similar moments of inertia (MI) for anterior-posterior bending (xMI) and lower for lateral bending (yMI) with a lower “shape-index” (yMI/xMI ratio) throughout, and higher resistance to buckling distally. These group differences were more evident in men and independent of group differences in bone mass. These results contrast with those observed in the tibia, where, as expected, structural indicators of bone strength were greater in trained than untrained individuals. Proximally, the larger lateral flexibility of runners' fibulae could improve the ability to store energy, and thereby contribute to fast-running optimization. Distally, the greater lateral fibular flexibility could reduce bending strength. The latter appears to have been compensated by a higher buckling strength. Assuming that these differences could be ascribed to training effects, this suggests that usage-derived strains in some bones may modify their relative structural resistance to different kinds of deformation in different regions, not only regarding strength, but also concerning other physiological roles of the skeleton.

## Introduction

The human tibia and fibula, despite being spatially close, experience substantially different loading environments during locomotion (1–5). The fibula's contribution to axial shank loading varies substantially with magnitude and position, from <5% of low-magnitude axial loads in ankle *varus* to about 19% during high-magnitude loading in dorsiflexion (6, 7). In addition, large differences were reported in structural behavior and stiffness/strain distribution along the human fibula subjected to varying loading configurations (8), and little is known about the transmission of bending and torsional forces in the shank. In fact, forces are transmitted in part through the tibiofibular and other ligaments (6) and the interosseous membrane, a mechanical contribution which has been scarcely investigated.

**Figure 1:**
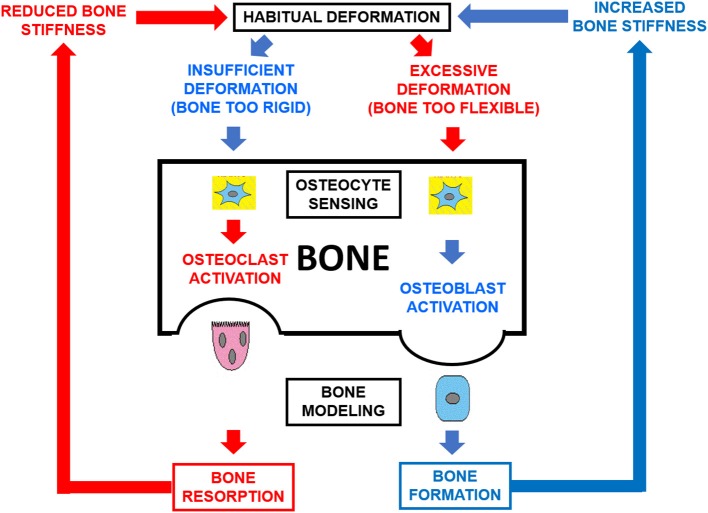
Schematic representation of the double-loop feedback system [bone mechanostat (8)] which controls the structural stiffness of bones as a function of the mechanical usage of the skeleton. Osteocytes sense the magnitude of the induced strains and modulate bone formation and destruction by osteoblasts and clasts (OB, OC) directionally in their environment. As a result, bone modeling is oriented tending to compensate for any directional inadequacy of bone structural stiffness.

Bones generally adapt to increased loads by slowly increasing in mass and/or by optimizing the distribution of the mass to increase the architectural efficiency of their design in the predominant directions determined by the history of their customary mechanical usage. Accordingly with the mechanostat Theory (9), the mechanism chronically involved in the bone response to loading is chiefly bone modeling (i.e., bone formation and destruction in different sites of the structure; Figure 1). As a result, the structure and strength of bones should reflect both their morphogenetical determination and mechanical environment (10, 11). Recent studies suggest that tibia and fibula, in addition to differing substantially in cortical structure, respond differently to the same kind of mechanical stimulation. In the human *tibia*, we have shown that (1) the cortical structure is highly adapted to compression stresses throughout the bone, with a smoothly variable adaptation to bending and torsion which reaches maximum effectiveness at the mid diaphysis (2), and (2) in long-distance runners, the pQCT-assessed cortical mass and diaphyseal design and strength indicators were all significantly larger than those of untrained controls, proportionally to the uniform variation of compression, bending and torsion stresses supported throughout the bone (3). In contrast, along the *fibula* shaft we have described no less than five different tomographic regions with varying structural features, suggesting that the adaptation of fibula structure to bending and torsion follows a non-uniform pattern along the shaft (5).

To note, the fibula appears to be little affected by long-term disuse, except in the epiphyseal sites (12, 13). This contrasts with the large deficits in mineralized mass and differences in distal diaphysis geometry evident in the adjacent tibia. It was also observed that the age-related decrease in muscle mass/strength in healthy men was associated to reduced tibia but not fibula cortical mass (14). In addition, the cross-sectional design of the normal human fibula diaphysis suggests to be more irregularly influenced along the bone by bending and torsional forces (as revealed by the distribution of the corresponding cross-sectional moments of inertia values) than by compressive loads (as revealed by the distribution of cortical area values) (5). Furthermore, conflicting findings about the structural responses of the fibula to exercise have been reported in the few studies to date (1, 15–18). The fibula was shown to be irresponsive to 9-month resistive exercise with whole-body vibration in postmenopausal women (15) and was structurally reinforced in torsion by only high-impact exercise loading in premenopausal women (1). In men trained in high-impact sprinting in whom only the site at the fibula mid diaphysis was scanned, no differences attributable to training on fibula structure or volumetric BMD distribution were reported (16). However, hockey players (who accelerate and turn with substantial dorsiflexion and eversion of the foot) had greater fibular strength than runners (17), and footballers showed a more robust cortical structure of the fibula in the supporting than in the kicking leg (18).

These observations suggest that, oppositely to the tibia, the adaptability of cortical fibula structure to mechanical environment could vary in magnitude, type, and even in direction, and may show either positive or negative responses to similar kinds of mechanical stimulation along the bone, with high site specificity, and perhaps in an unpredictable way in some instances (19). Cristofolini et al. (8) showed different structural responses of the fibula and other leg bones to opposite regimens of stimulation which could not be explained by the theory of elasticity, and proposed that bone tissue could show a non-symmetric behavior in some instances, perhaps defying some aspects of Wolff's Law and mechanostat Theory (9). Others' observations (20–24) would be in consonance with that view. In fact, some fibula adaptations to different kinds of mechanical stimulation could have both positive and negative impact on the function. For example, a fibula that was more compliant to lateral bending could be somewhat weaker, but also more efficient at storing energy from muscle contractions for jumping (25). The question seems to be, how much fibular deformability should be allowed in order to favor the biomechanical performance of the bone without substantially increasing the risk of fracture.

The few (cross-sectional) available studies of exercise effects on the *fibula* have investigated only one or no more than 4 sites or only one sex. No study has described the responses to exercise throughout the male and female fibula, allowing comparison of regions which biomechanical and tomographic descriptions would suggest experience substantially different mechanical loading. To partially fill this gap, this study (also cross-sectional) aimed to describe differences in fibula structure as assessed by pQCT in trained long-distance runners and untrained controls of both sexes and compare them with those observed in the adjacent tibiae. The working hypothesis was that, in contrast with the tibia, the runners' fibula structure should show some regional differences with respect to that of the untrained individuals which could be more related with the functional behavior of the bone in running (with a high selective relevance) than with an improvement in bone strength.

## Materials and Methods

### The Study Participants

The studied individuals were 60 healthy, freely active, young white adults (30/30 men/women), residents of the urban area of Rosario City, Argentina. None of them had a history of fractures or diseases, smoking or drinking, or treatments affecting the skeleton, and none of the women had a history of menstrual disorders.

From that selected group, 30 individuals (15/15 men/women) aged 25–38 years had been engaged as a single, voluntary and self-controlled group in regular long-distance running comprising 3–5 sessions per week, 10–16 km per session, for 8–11 years, at an average velocity of 11.2 ± 0.7 km/h for men and 10.3 ± 0.7 km/h for women (runners group) until the time of the study. In a unique, cross-sectional observation, they were compared with a control group of 15/15 men/women of comparable age. The latter were selected from a freely recruited sample of voluntary participants after a public announcement made in the ambit of the UGR (i.e., within the same social environment as that of the runners), avoiding inclusion of those with excessive large or small weight and stature within sexes with respect to the corresponding means and SDs of the trained groups in order to minimize the influence of allometric associations of the assessed variables which could have been difficult to neutralize by the adjusting procedures applied. All individuals studied performed a similar, regular pattern of daily activities concerning activity at work, travel to and from places of work, and recreational activities which was equivalent to the “moderate” level of activity established by the *Global Physical Activity Questionnaire (GPAQ)* (26). The control groups had never been trained in running or in any other discipline involving a specific use of the legs; hence, they were regarded as active (non-sedentary) untrained individuals. The age and anthropometric data of the samples and their tibia length (as assessed for the tomographic studies—see below) and tibia length/body height ratio are given in Table 1. The statistical significance of the inter-group differences within sexes in all the above variables is also indicated as a measure of the degree of homogeneity achieved for the respective samples.

**Table 1:** Means and SDs of age, body weight, body height, body/mass index, tibia length, and tibia length/body height ratio of the studied groups, and ANOVA tests of the differences in these variables between sedentary and runner individuals within each sex.

	**Men**			**Women**		
	**Untrained**	**Runner**	**ANOVA, *p***	**Untrained**	**Runner**	**ANOVA, *p***
Age, year	30.8 ± 3.0	32.7 ± 3.0	0.382 (ns)	30.4 ± 2.9	30.8 ± 3.4	0.173 (ns)
Weight, kg	78.1 ± 6.3	74.3 ± 5.	0.589 (ns)	57.6 ± 5.7	54.1 ± 4.1	0.262 (ns)
Height (h), cm	173.9 ± 3.3	173.2 ± 3.1	0.400 (ns)	163.5 ± 3.1	161.0 ± 4.1	0.200 (ns)
Body/mass index	26.8 ± 4.2	26.4 ± 1.4	0.748 (ns)	21.8 ± 0.8	22.3 ± 1.2	0.180 (ns)
Tibia length, mm	39.8 ± 1.8	39.2 ± 2.1	0.401 (ns)	37.2 ± 2.3	36.5 ± 2.1	0.199 (ns)
Tibia length/h ratio	0.22 ± 0.01	0.23 ± 0.0	0.503 (ns)	0.23 ± 0.01	0.22 ± 0.01	0.161 (ns)

Informed consent was obtained by every individual before inclusion in the study. The study was approved by the Hospital's Ethics Committee (*Comité de Ética, Hospital Provincial del Centenario*, Rosario, Argentina).

### pQCT Measurements

An *XCT-2000* scanner *(Stratec, Germany)*, software version 5.0, was used to scan the entire dominant leg of each individual. The radiation dose was about 0.9 μSV per scan (<20 μSv for the whole study). The slices were 2.5 mm thick, and the in-plane pixel size was 0.5 mm. A previously reported, computer-aided procedure to serially scan the whole tibia (2) was used to analyze the corresponding length of the adjacent fibula. Leg scans were obtained at every 5% of the leg length from the projection of the tibia-talar joint line to the articular line of the knee. Scans were numbered from S5 (5% site, located 5% of the scanned length proximal to the tibia-talar joint) to S95 (95% site, located 95% of the scanned length proximal to the tibia-talar joint). The device allows for no more than 9 slices per session. Thus, each half of the scanned length of the leg had to be studied separately and the scan at S50 (starting point for scanning the proximal segment of the leg) could not be obtained. The distal end of the fibula (analogously to the tibia malleolus as a distal landmark) could not be scanned below the S5 because the field size did not allow for introduction of the foot. Therefore, a total of 18 scans were obtained per each fibula and tibia, and hitherto any reference to the studied bones applies to the described length taken proximally from S10 to S80 (i.e., 14 scans per individual) in merit of accuracy and reliability of the measurements or calculations. Threshold values for total and cortical bone were selected at 180.0 and 710.0 mg/cm^**3**^, respectively, using the parameters *contmode* 2, *peelmode* 2, and *cortmode* 1. The following indicators were obtained as allowed in every site studied.

#### Cortical Perimeters and Thickness

- *Periosteal perimeter*, in mm.
- *Endocortical perimeter*, in mm.
- *Cortical thickness:* average thickness of the bone cortex automatically given by the machine, in mm.

These indicators describe the most elementary geometric parameters which are directly affected by bone modeling.

#### Bone “Mass” Indicator

- *Cortical bone area*, in mm^2^.

This indicator reflects the amount of available cortical tissue in the bone section. It was studied as such and was also normalized by body mass (27) to evaluate the influence of allometric factors in the determination of the observed differences.

#### Bone Tissue Mineralization [and Intrinsic Stiffness (28)] Indicator

- *Volumetric cortical mineral density (cortical vBMD)* = *cortical BMC (mg/mm of scan thickness)/cortical area*, in mg/cm^3^ [data shown for only the S15-S75 range of bone sites as allowed by the cortical thickness (29)].

This indicator assesses the degree of mineralization of bone tissue, which is regarded as an indirect indicator of its intrinsic stiffness (elastic modulus) (28).

#### Indicators of the Architectural Efficiency of Cortical Tissue Distribution Within the Bone Section

- *Cross-sectional moments of inertia (MI's)*: The reference axes for MI calculation were the ML (x) axis (AP bending MI, xMI), and the AP (y) axis (ML bending MI, yMI), in mm^4^. Total sums of products of the area of every cortical pixel by its squared perpendicular distance to the x and y axes of the image center of mass were obtained, after rotating the axis system until achieving a maximal “y” value of the AP axis. The xMI and yMI values are proportional to the stiffness of bone shafts in AP and ML bending, respectively. All MI values were studied after being normalized by the product of body weight times the bone length (bw^*^L) (27) to minimize their allometric associations.
- *Fibula/tibia MI ratio*. The yMI was also expressed as the ratio between its fibula and tibia values as a further, allometrically-free comparison of yMI values between the two bones.
- *yMI/xMI ratio [“shape index”* (30), dimensionless]: relationship between the yMI and xMI values determined at each bone site. This index is regarded as a *body size-unrelated* indicator of the relative development of the yMI with respect to that of the xMI in the same bone sites or regions.
- *Buckling ratio (BR)* = *R/CtTh* (dimensionless), being R the mean diaphyseal cross-sectional radius, and CtTh the average cortical thickness. This indicator is proportional to the *risk* of the diaphysis to fail in buckling.
- *Buckling Resistance Index (BRI)* = 1/BR (dimensionless). In this study, this index is regarded as an indicator of the *resistance* of the diaphysis to fail in buckling (31).

### Statistical Analyses

*Statistica (StatSoft Inc., USA, 2008)* software was used. Means and SEs were calculated for each indicator separately in men and women and in runners and controls within each sex and plotted by scanned site for each bone. The distribution of all the pQCT indicators throughout the fibula was examined in order to define site-specific differences throughout the bone, and to compare them with those observed for the tibia. Factorial ANOVA of the evolution of the studied indicators in men and women in every site throughout the two bones (“site-effect”) evaluated the higher-order interactive effects of all the studied groups (“sex effect” and “training effect”). The procedure automatically detected any group of successive sites within which the “training effect” showed significant global differences between trained and untrained individuals within each sex. No statistically significant differences were detected for single, isolated sites in any instance. Thus, continuous segments of bone diaphyses showing significant results could be objectively defined for each sex and selected for further comparisons.

The numbers of individuals per group were all larger than those which were analyzed in all our 4 previously published studies employing the same analytical model (2–5). The potency of the method for comparisons between runner and untrained individuals within each sex for α = 0.05 ranged from 0.84 for a defined significant segment containing a minimum of 3 consecutive sites to a minimum of 0.99 when 7 or more consecutive sites were included in the selected segment.

## Results

Table 1 shows the relative homogeneity of the trained and untrained samples concerning age and some of the more relevant anthropometric features to the biological determination of all bone parameters studied.

**Figure 2:**
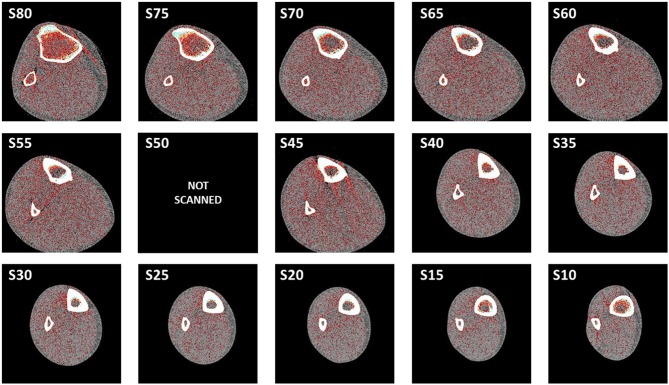
Examples of the 14 selected scans corresponding to the leg of one of the untrained individuals, taken from the most proximal site (S80) to the most distal one (S10), as described in Materials and Methods.

Figure 2 shows the complete series of 14 selected scans taken from the leg of one of the untrained individuals, from the most proximal (S80) to the most distal (S10) site.

The comparison of runner vs. control data differed from tibia to fibula indicators. The differences observed were generally more evident in men than in women and exhibited some bone-site specificity in several cases, as described below.

### Bone Mass Indicator (Cortical Bone Area—Figure 3A)

In the tibiae (left), cortical area was significantly higher in runners than controls throughout the bone in men and along the central-proximal region in women, while in the fibulae (right), cortical area was lower in runners than controls in the proximal (men) or central-proximal regions (women). Adjustment of the data to body mass (not shown) did not affect the intra- or inter-group behavior of the data.

**Figure 3:**
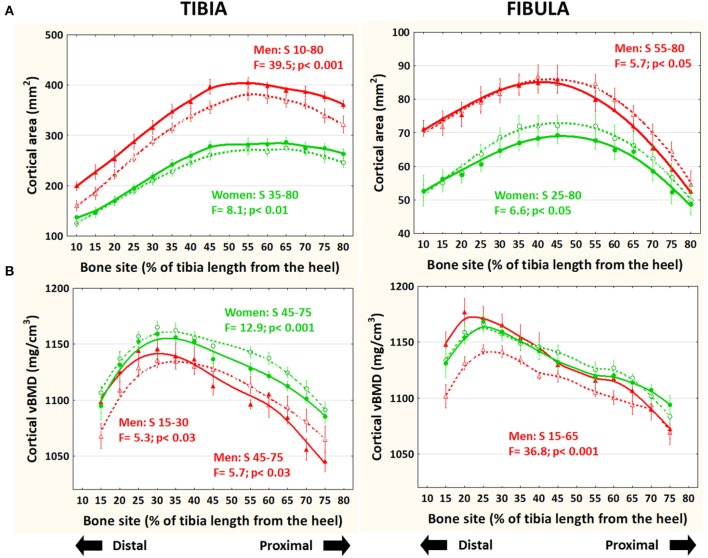
Distribution of means and S.E. of the cortical bone cross-sectional area **(A)** and volumetric mineral density **(B)** of the tibia (left graphs) and fibula (right graphs) of runner (continuous lines) and untrained (dashed lines) men (red curves) and women (green curves) in all studied sites along the bones. Statistical significances of the factorial-ANOVA assessed differences between runners and untrained individuals within each sex and the corresponding, automatically defined site intervals showing significant results are indicated. Outside the indicated sites these differences were non-significant.

### Bone Mineralization and Tissue Stiffness (28) Indicator (Cortical vBMD—Figure 3B)

In the tibiae, cortical vBMD was slightly but significantly lower proximally (−1% to −3%) in runner men and women and higher distally (+1 to +4%) in runner men with respect to controls. In the fibulae, cortical vBMD was significantly higher (+2 to +5%) in runner men (only) than in controls below S70.

### Cortical Perimeters and Thickness (Figure 4)

**Figure 4:**
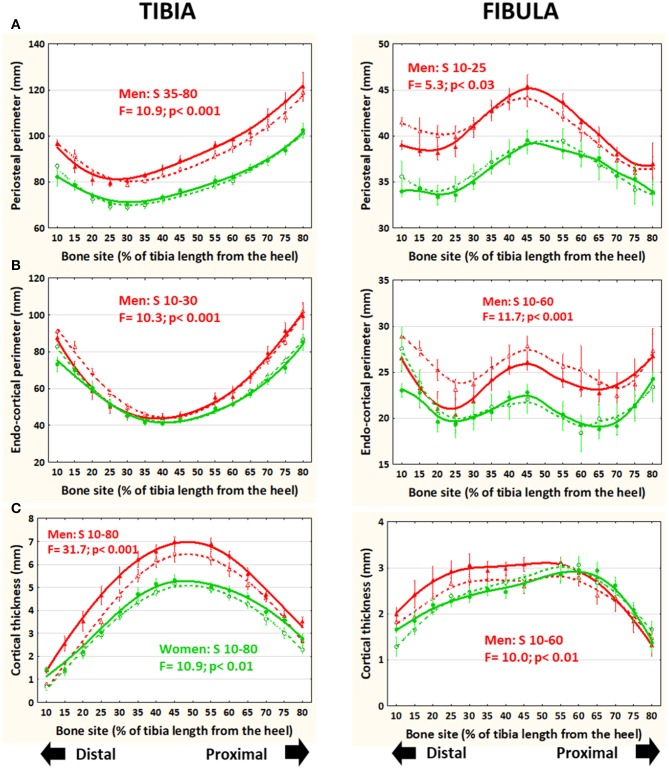
Distribution of means and S.E. of the cross-sectional periosteal **(A)** and endocortical **(B)** perimeters and cortical thickness **(C)** of the tibia (left graphs) and fibula (right graphs) of runner (continuous lines) and untrained (dashed lines) men (red curves) and women (green curves) in all studied sites along the bones. Statistical significances of the factorial-ANOVA assessed differences between runners and untrained individuals within each sex and the corresponding, automatically defined site intervals showing significant results are indicated. Outside the indicated sites these differences were non-significant.

In running men only, periosteal perimeter was significantly larger at the central-proximal region of the tibia and slightly but significantly smaller toward the distal end of the fibula with respect to untrained controls (Figure 4A). These differences were largely reduced or neutralized after adjustment by body weight (not shown). The endo-cortical perimeter was significantly smaller in runners than in control men and women in the distal tibiae and in the central-distal fibulae only in the men (Figure 4B). The balance of these differences led to a significantly higher cortical thickness in runners than controls all along the tibiae in both sexes but only in the central-distal fibulae in men's bones (Figure 4C).

### Bone Cross-Sectional Design Indicators (MIs, “Shape Index,” BRI—Figures 5, 6)

**Figure 5:**
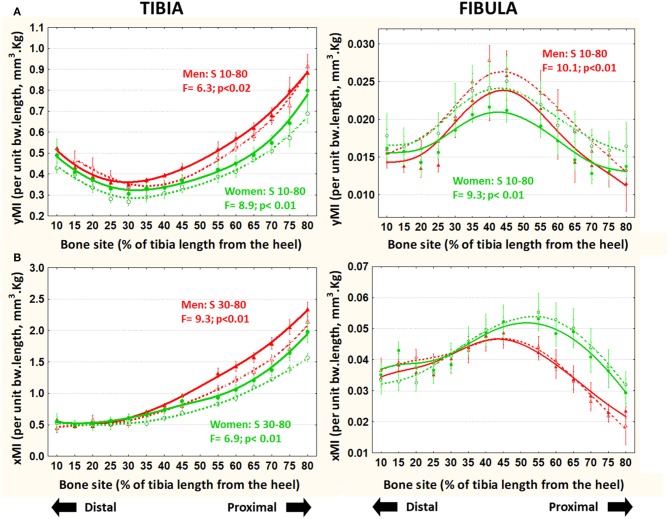
Means and S.E. of the [bw*L]-adjusted cross-sectional moments of inertia for ML bending (yMI, **A**) and AP bending (xMI, **B**) of the tibia (left graphs) and fibula (right graphs) of runner (continuous lines) and untrained (dashed lines) men (red curves) and women (green curves) in all studied sites along the bones. Statistical significances of the factorial-ANOVA assessed differences between runners and untrained individuals within each sex and the corresponding, automatically defined site intervals showing significant results are indicated. Outside the indicated sites these differences were non-significant.

In the tibiae, both [bw^*^L]-adjusted MIs were progressively larger in runners than in controls, proximally to S30 in men and to S10 in the women. In contrast, in the fibulae, the adjusted yMI and xMI varied differently. While the yMI was significantly *lower* in runner than in control men and women virtually throughout the bone (Figure 5A), the xMI showed no significant differences (Figure 5B).

**Figure 6:**
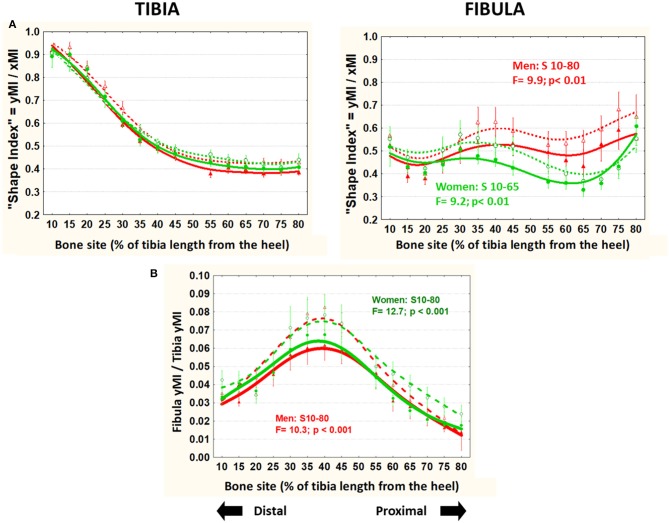
Means and S.E. of the “shape index” (unadjusted yMI/xMI ratio) of the tibia (left graph) and fibula (right graph) **(A)** and the fibula/tibia yMI ratio **(B)** of runner (continuous lines) and untrained (dashed lines) men (red curves) and women (green curves) in all studied sites along the bones. Statistical significances of the factorial-ANOVA assessed differences between runners and untrained individuals within each sex and the corresponding, automatically defined site intervals showing significant results are indicated. Outside the indicated sites these differences were non-significant.

The body-size unrelated “shape index” (unadjusted yMI/xMI ratio; Figure 6A) showed virtually no variation in the tibiae in all groups throughout. Instead, in the fibulae, it was generally higher in men than in women in the central-proximal region, and significantly lower in runners than controls all through (average −18% in men and −8% in women).

The ratio between fibula and tibia yMIs (unrelated to body size) was lower in all runners than controls throughout the bones (Figure 6B).

**Figure 7:**
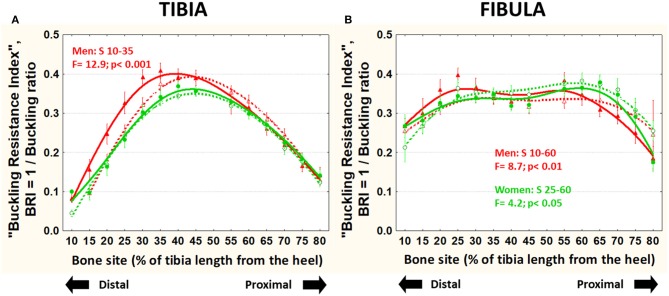
Means and S.E. of the “buckling resistance index,” BRI = 1/buckling ratio) of the tibia (left graph) **(A)** and fibula **(B)** (right graph) of runner (continuous lines) and untrained (dashed lines) men (red curves) and women (green curves) in all studied sites along the bones. Statistical significances of the factorial-ANOVA assessed differences between runners and untrained individuals within each sex and the corresponding, automatically defined site intervals showing significant results are indicated. Outside the indicated sites these differences were non-significant.

The BRI (also unrelated to body size) was significantly higher in runner than control men (central-distally in both bones) and women (only in the distal fibula) (Figure 7).

## Discussion

The aim of this cross-sectional study was to describe differences in fibula cortical bone structure between trained runners and controls throughout the bone's length, and to compare these differences with those observed in the neighboring tibia of the same individuals. We observed site-specific differences in several cortical bone parameters of the fibula, with values in trained individuals being generally similar to or lower than those observed in controls with the exception of higher resistance to buckling distally. These results contrast with those observed in the tibia, where structural indicators of bone strength were generally greater in trained individuals.

It is widely accepted that the long-term mechanical stimulation of any mobile bone strengthens rather than weakening its structure, and *vice-versa* (32). In this study, the differences observed between the runners' tibiae were congruent with that idea, as expected after a previous observation (3). However, the fibula data revealed (1) a different behavior of medio-lateral and A-P bending stiffness indicators, yMI and xMI, in runners than in controls, and (2) a different behavior of the inter-group differences in yMI (decrease in runners) and BRI (increase in runners) toward the distal end of the bone. These differences were generally more evident in men's than in women's bones, possibly because of the differences in muscle force and hormonal status which may exert direct effects on bones that are known to show some skeletal-envelope and site specificity (33–36).

The distribution of the observed inter-group differences throughout each bone was also contrasting. In fact, in the runners' tibiae, the differences in the indicators of bone AP and ML bending stresses (xMI, yMI) increased consistently in parallel in the proximal direction, progressively improving the ability of the cross-sectional design to resist the natural, training-induced stresses in bending and torsion with respect to untrained controls, as previously described (3). In contrast, in the runners fibulae, each of the two MIs and also the BRI changed differently through the bone, suggesting that, in individuals subjected to the same training, the fibula can show either a higher or a lower bone stiffness than that of untrained individuals, depending on the bone region and kind of stress considered (ML or AP bending, buckling).

In addition, whatever the nature of the underlying mechanisms involved, the differences in fibula indicators between runners and controls were associated to some apparent incongruence between the differences observed in fibula mass/density and in design/strength indicators, chiefly derived from regional differences in the periosteal and endosteal perimeters. In fact, in the central-distal region of runner's fibulae, despite that bone mass was similar, cortical thickness was greater than in controls. Furthermore, the ML bending stiffness (as assessed by the yMI) was lower than controls throughout the bone, while the A-P bending stiffness (as assessed by the xMI) was similar in both groups, independently from the regional differences in bone mass. This contrasts with the generally higher values of all mass and design/strength indicators observed in the tibiae of the same individuals in this and in a similar, previously studied sample (3). Therefore, the observed differences in fibula MIs development should have reflected *differences in proximal and distal behavior* of bone modeling and/or remodeling.

The larger resistance to buckling (BRI) observed distally in runners' fibulae than in controls' can be also related to differences in all bone mass, design and mineralization. In fact, both the BRI (proportional to cortical thickness) and cortical vBMD (a general correlate of bone tissue stiffness) were higher in the runners in that region. There is some evidence that these differences, biomechanically opposite to the negative differences observed in ML bending stiffness (yMI), may be explained by a larger sensitivity to mechanical loading in the endocortical than in the periosteal surface (37) as determined by systemic (non-directional) factors (the “*anti-mechanostat”*) (38).

The above proposals to explain the contrasting behavior of different indicators in our runners' fibulae [in agreement with the striking structural differences between bones which were already observed by us in chronically immobilized legs (13)] are congruent with our description of five morphologically different regions throughout the human fibula in which some structural indicators varied widely between sites (5). This suggests that the bone could respond differently to different kinds of strains with large site specificity. The observation of different responses to exercise of structural indicators of bones situated in the same limb or in different regions of the same bones is not new (20, 23). The nature of some exercise-induced changes has been shown to be negative in some instances, with a possible association with a locally reduced osteoblast generation or activity (22, 39).

At any rate, these findings are congruent with the current conception that the “customary strain level” to which bone tissue seems to be adapted is not constant, but varies by skeletal location, type of strain, strain gradient, and loading history (19, 33, 34, 40–43). In the fibula, running could have induced repetitive loads in AP and ML bending (17, 44) and many factors (not assessed in this study) could have affected the type of strain induced to the fibulae by long-distance running, including the behavior of the ankle joint and the distal tibiofibular syndesmosis, and the stiffness of the interosseous membrane (6, 45).

If the above assumptions are right, then our results would also suggest that the behavior of the fibula in our chronically trained runners would lay beyond our traditional knowledge about bone responses to mechanical environment as managed by the bone *mechanostat* (9). In consonance with this interpretation, cross-sectional studies in animals showed that loading-induced bone growth may not be directionally related to the induced local strains in every instance (24, 46).

The above interpretations are congruent with some phylogenetical observations. In fact, the evolutionary pathways of the fibula oscillate from region-specific robustness or slenderness according to survival needs even in taxonomically close species, including hominoids (17, 45). Following that idea, in can be proposed that, in our runners' fibulae, the generally larger compliance to ML bending with respect to controls might conveniently improve the ability of the bone to store elastic energy during the contraction of the locally inserted muscles which act on the foot during running/jumping (25, 47–49). On the other hand, the weakening of the proximal and distal design of the fibula concerning ML bending could increase the risk of ML-bending fractures. However, this apparent inconvenience could be at least partially overcome by the distal enhancement of buckling strength which was also observed. This could reduce the risk of most common (buckling) fractures at the most critical [distal (50)] region of the bone.

Therefore, in congruence with our hypothesis, our findings could be more easily explained if the fibula *could respond distinctly to different types of strains in different regions*, as observed in the ulna (19, 51) and in *in-vitro* studies at the cellular level of biological organization (52). As stressed by Ruff (19), (1) “strain distributions in bones that are less specialized for cursorial locomotion more closely match traditional expectations of greater bone strength in directions of higher strain, especially during vigorous movement” (51), and (2) “some degree of bending could actually be beneficial to bone tissue by maintaining strains within the ‘optimum customary’ window (53) avoiding potentially catastrophic strains in ‘unusual’ orientations,” as observed in this study. Thus, *bone structure may be genetically designed in some cases to confine strains to more predictable patterns rather than strictly to minimize strains* (48, 53, 54).

In other words, our results would suggest that the fibula could be regarded as a “less predictable” bone than the tibia, the reasons for the difference being as much phylogenetic as mechanical (19). This interpretation is congruent with the assumption that, *rather than mathematical optimization rules for bone architecture, there seems to be just a biological process which adapts bone structure to mechanical demands, adequate for evolutionary endurance* (55, 56).

## Limitations of the Study

The cross-sectional nature of the study precludes any reference to “improvements” or “impairments” of the studied indicators as directly derived from training, thus restricting the discussion of the observed effects to simple comparisons between the bone features of the studied groups. In fact, results could, at least partly, be explained by self-selection bias, given that the athletes chose their disciplines by their own volition. However, the observed anthropometric homogeneity of the samples, the MI adjustments to body weight and bone length, the behavior of the size-unrelated “shape index,” and the comparisons made between fibula and tibia MI data of the same individuals should have minimized any interference from allometric correlates with the described inter-group differences. Therefore, the different behavior of cortical bone mass distribution in tibia and fibula between runners and controls could be reasonably regarded as a “training effect.” Nevertheless, we think that further, specifically designed longitudinal studies will be needed to confirm our findings and conclusions.

The number of individuals per group (15), although being somewhat larger than those selected for our previous, similar studies (1–5), could be regarded as relatively small. However, the acceptable potency of the study and the statistical strength of most of the differences observed would support our interpretation of the reported results. At any rate, further studies with larger number of individuals (and wider ranges of ages) will be needed to support our interpretation of the present findings.

The studied sample comprised only healthy, active (not sedentary) adult men and pre-menopausal women that were either untrained or trained in long-distance running for several years. Thus, the conclusions should be restricted to these specific experimental conditions.

The study model was restricted to determinations of bone features which can be assessed by pQCT, i.e., to only those which are strictly derived from and affected by changes in bone mineralization and geometry of the studied bones.

## Conclusions

This study affords some original evidence of a striking, “non-canonical” behavior of the human fibula concerning its structural response to mechanical environment as compared to the adjacent tibia. In the tibia, runners were found to have generally bigger, stronger diaphyses. In the fibula, however, runners exhibited diaphyses which were somewhat smaller in size and lower in structural strength compared to non-runners.

Those differences, more evident in men than in women, could have enhanced the ability of the fibula to contribute to fast-running optimization, in spite of a general weakening of the bone in lateral bending and independently of the differences observed in bone mass. However, the buckling strength of the bones seemed to have been conveniently improved at its distal end, which is the site most prone to fracture.

These findings support the idea that bone functional adaptation is complex and not easy to predict based on our current understanding of the process. The study suggests that mechanical loading may affect different bones in distinct ways, beyond the scope currently proposed by the mechanostat Theory, not only regarding resistance to fracture, but also concerning other bone features which may show some selective connotations.

## Data Availability Statement

The datasets generated for this study are available on request to the corresponding author.

## Ethics Statement

The studies involving human participants were reviewed and approved by Comité de Ética, Hospital Provincial del Centenario, Rosario, Argentina. The patients/participants provided their written informed consent to participate in this study.

## Author Contributions

RC, GC, and JF contributed to conception and design of the study. LP and NP were responsible for data collection. SL and LN were responsible for data and statistical analysis. JF wrote the first draft of the manuscript. JR, AI, and JF provided interpretation of the results. JF and AI wrote sections of the manuscript. All authors contributed to manuscript revision, read and approved the submitted version.

### Conflict of Interest

The authors declare that the research was conducted in the absence of any commercial or financial relationships that could be construed as a potential conflict of interest.
